# Supplementary material for: AI-enhanced collective intelligence
Source: Patterns (N Y). 2024 Oct 10;5(11):101074. doi: 10.1016/j.patter.2024.101074 (PMC11573907; doi:10.1016/j.patter.2024.101074)
Supplement: Table S1. Main concepts of key terms in human-AI CI [file mmc1.pdf]

Patterns, Volume *5*

Supplemental information

AI-enhanced collective intelligence

Hao Cui and Taha Yasseri

Term	Main concepts	References
AI-enhanced Collective Intelligence	A system where artificial intelligence tools and techniques are integrated to augment the collective intelligence of a group, improving decision-making, problem-solving, and innovation.	
Complex Adaptive System	A subset of complex systems characterized by the ability of its components to adapt and learn from interactions with each other and the environment, leading to dynamic evolution.	[60]
Collective Intelligence	The group intelligence that emerges from the collaboration and collective efforts of individuals working together towards a common goal.	[2]
Complex System	A system composed of interconnected parts that exhibit collective behavior, self-organization, and adaptation to changing environments, often displaying emergent properties.	[49]
Complexity Theory	A framework for understanding how interactions among components of a system give rise to collective behaviors and emergent phenomena that cannot be predicted from individual parts.	[49]
Emergence	The process through which larger patterns, structures, or behaviors arise from the interactions among smaller or simpler entities that do not exhibit such properties individually.	[60]
Hybrid Intelligence	The combination of human intelligence and artificial intelligence, leveraging the strengths of both to solve complex problems more effectively.	[34]
Multilayer Network	A network model where nodes represent entities and different layers represent different types of interactions or relationships between these entities.	[56]
Wisdom of Crowds	The collective opinion of a diverse and independent group of individuals can be more accurate than that of a single expert, leveraging diversity of thought and decentralization.	[10]

Table S1: Main concepts of key terms in human-AI collective intelligence. For references, see the main article.
