# Supplementary material for: AI-enhanced collective intelligence
Source: Patterns (N Y). 2024 Oct 10;5(11):101074. doi: 10.1016/j.patter.2024.101074 (PMC11573907; doi:10.1016/j.patter.2024.101074)
Supplement: Table S2. Summary of CI and AI aspects of various application examples [file mmc2.pdf]

Application	Area	Aspects of Collective Intelligence	Aspects of Artificial Intelligence
Safecity	Public sector, NGO	• Crowdsourced reporting<br>• Community discussion and initiatives	• Predictive analytics<br>• Sentiment analysis
Ushahidi	Public sector, NGO	• Crowdsourced crisis information<br>• Community reporting	• Machine learning<br>• Natural language processing
Bluenove	High Tech (software)	• Open consultation<br>• Collaborative ideation	• Natural Language Processing<br>• Emotion analysis
Figure Eight	High Tech (software)	• Crowdsourced annotation	• Machine learning<br>• Automated Workflows
Civil War Photo Sleuth	Media, telecommunication, entertainment, hospitality	• Crowdsourced Identification<br>• Collaborative research	• Facial recognition<br>• Image analysis
Bellingcat	Media, telecommunication, entertainment, hospitality	• Crowdsourced investigation<br>• Community collaboration	• Multi-modal data analysis<br>• Pattern recognition
Litterati	Energy, natural resources	• Crowdsourced litter data<br>• Community engagement	• Computer vision<br>• Predictive modeling
eBird	Energy, natural resources	• Crowdsourced bird observations<br>• Peer review and validation	• Computer vision<br>• Predictive modeling
Kialo Edu	Education and academia	• Structured debates and discussions	• Natural language processing<br>• Sentiment analysis<br>• content recommendation
Zooniverse	Education and academia	• Crowdsourced research<br>• Collaborative data analysis	• Machine learning classification<br>• Pattern recognition
Human Dx	Healthcare	• Crowdsourced medical expertise<br>• Collaborative problem-solving	• Pattern recognition<br>• Data analytics
CrowdEEG	Healthcare	• Collaborative annotation	• Machine learning training<br>• Predictive modeling
Numerai	Financial services	• Crowdsourced predictions<br>• Community collaboration	• Ensemble Learning<br>• Predictive Analytics
CryptoSwarm AI	Financial services	• Crowdsourced market insights<br>• Swarm intelligence<br>• Community feedback	• Swarm AI<br>• Predictive modeling<br>• Automated alerts

MarineTraffic	Supply Chain, Real Estate	• Crowdsourced Vessel Tracking<br>• Community Contributions<br>• Data Sharing	• Automatic Identification System (AIS) Data Analysis<br>• Object detection<br>• Predictive Analytics
Waze	Supply Chain, Real Estate	• Crowdsourced Traffic Data<br>• Community Editing and feedback	• Route Optimization<br>• Personalized Recommendations<br>• Voice assistant integration<br>• Predictive Analytics
WeFarm	Agriculture	• Crowdsourcing advice<br>• Knowledge sharing<br>• Problem-solving	• Automated matching<br>• Personalized recommendations<br>• Natural language processing
Mercy Corps AgriFin	Agriculture	• Community-driven peer learning<br>• Knowledge sharing	• AI imagery<br>• Data analytics and modeling<br>• Recommendation algorithm

Table S2: Summary of CI and AI aspects of various application examples.
